# Validity of the posttraumatic stress disorders (PTSD) checklist in pregnant women

**DOI:** 10.1186/s12888-017-1304-4

**Published:** 2017-05-12

**Authors:** Bizu Gelaye, Yinnan Zheng, Maria Elena Medina-Mora, Marta B. Rondon, Sixto E. Sánchez, Michelle A. Williams

**Affiliations:** 1 Department of Epidemiology, Harvard T. H. Chan School of Public Health, 677 Huntington Ave, K505F, Boston, MA 02115 USA; 2 National Institute of Psychiatry, Mexico City, Mexico; 3 Department of Medicine, Cayetano Heredia Peruvian University, Lima, Peru; 4 Universidad Peruana de Ciencias Aplicadas, Lima, Peru; 5 Asociación Civil PROESA, Lima, Peru

**Keywords:** Post-traumatic stress disorder; PCL-C, Pregnancy, Psychometrics, Peru

## Abstract

**Background:**

The PTSD Checklist-civilian (PCL-C) is one of the most commonly used self-report measures of PTSD symptoms, however, little is known about its validity when used in pregnancy. This study aims to evaluate the reliability and validity of the PCL-C as a screen for detecting PTSD symptoms among pregnant women.

**Methods:**

A total of 3372 pregnant women who attended their first prenatal care visit in Lima, Peru participated in the study. We assessed the reliability of the PCL-C items using Cronbach’s alpha. Criterion validity and performance characteristics of PCL-C were assessed against an independent, blinded Clinician-Administered PTSD Scale (CAPS) interview using measures of sensitivity, specificity and receiver operating characteristics (ROC) curves. We tested construct validity using exploratory and confirmatory factor analytic approaches.

**Results:**

The reliability of the PCL-C was excellent (Cronbach’s alpha =0.90). ROC analysis showed that a cut-off score of 26 offered optimal discriminatory power, with a sensitivity of 0.86 (95% CI: 0.78–0.92) and a specificity of 0.63 (95% CI: 0.62–0.65). The area under the ROC curve was 0.75 (95% CI: 0.71–0.78). A three-factor solution was extracted using exploratory factor analysis and was further complemented with three other models using confirmatory factor analysis (CFA). In a CFA, a three-factor model based on DSM-IV symptom structure had reasonable fit statistics with comparative fit index of 0.86 and root mean square error of approximation of 0.09.

**Conclusion:**

The Spanish-language version of the PCL-C may be used as a screening tool for pregnant women. The PCL-C has good reliability, criterion validity and factorial validity. The optimal cut-off score obtained by maximizing the sensitivity and specificity should be considered cautiously; women who screened positive may require further investigation to confirm PTSD diagnosis.

**Electronic supplementary material:**

The online version of this article (doi:10.1186/s12888-017-1304-4) contains supplementary material, which is available to authorized users.

## Background

Posttraumatic Stress Disorder (PTSD) is a common and debilitating trauma and stressor-related disorder that occurs following exposure to life events involving real or threatened death, severe injury or sexual assault [1]. PTSD is characterized by four symptom clusters: re-experiencing, avoidance of stimuli associated with the event, negative alterations in cognition and mood, and physiological hyperarousal and reactivity [1]. Changes to the criteria for PTSD have been introduced in the fifth Diagnostic and Statistical Manual of Mental Disorder (DSM-5) definition including moving PTSD from the anxiety disorders category to a new class of trauma- and stressor-related disorders. In addition, the three symptom clusters in the fourth edition of the DSM (DSM-IV) [2] were expanded into four by splitting the avoidance and numbing cluster into an avoidance cluster and negative alterations in cognitions and mood cluster. In the United States, the lifetime prevalence of PTSD in the general population ranges from 6 to 8% and ranges from 1 to 9% worldwide [3–6].

Women have a greater risk of PTSD as compared with their male counterparts [3, 4, 7–9]. Of note, women are at particularly elevated risk for PTSD during their reproductive years, especially during pregnancy and postpartum, with PTSD symptoms likely to reach their peak severity closer to delivery [10]. The point prevalence of PTSD among pregnant women has been reported to range from 2.3 to 16% [7, 11–13]. This wide variation has been attributed to differences in the level and amount of trauma exposure, proximity to the trauma, length of time since trauma, differences in assessment tools used and population characteristics [7, 11–13]. PTSD in pregnancy is associated with perinatal complications including ectopic pregnancy [14], spontaneous abortion [14], hyperemesis gravidarum [14], preterm labor [15], preterm delivery [16, 17], low birth weight [16] and excessive fetal growth [14]. PTSD during pregnancy may also lead to other psychiatric disorders, including first-onset major depression, anxiety, bipolar disorder and alcohol abuse or dependence [8]. However, diagnosis and treatment of PTSD among pregnant and postpartum women remains low, especially in low- and middle-income countries. This is due, in part, to the lack of skilled mental health professionals, stigma associated with mental disorders, and lack of cross-culturally validated screening and diagnostic instruments [18–22].

The Posttraumatic Stress Disorders Checklist (PCL), one of the most commonly used self-reported questionnaires for PTSD detection [23], consists of 17 items which correspond to the symptoms of PTSD in the DSM-IV [24]. The Civilian version of PCL (PCL-C), designed for general traumatic experience [25], has been shown to be a valid and reliable tool for screening and managing PTSD symptoms in various clinical and population-based settings according to DSM-IV criteria [26, 27]. Recently, the PCL was revised to PCL-5 reflecting DSM-5 changes to the PTSD criteria. Overall, the symptoms of PTSD have been reported to be comparable between DSM-5 and DSM-IV [28].

Despite its increased utility, to date, no study has evaluated the utility of PCL-C in pregnancy. Moreover, only four investigative teams have published studies documenting the psychometric properties of PCL-C when used in South American populations [29–32]. Notably, none of these studies were conducted in Peru. Peru, a middle-income and post-conflict country, is one of the countries with the highest prevalence of violence against women [33]. A recent study conducted in Peru found that one in four women reported being physically or sexually abused during pregnancy (21.4%), including in extreme forms of violence such as rape [34]. Furthermore, Barrios et al. recently noted that 24% of pregnant women reported being raped or being victims of attempted rape before the age of 15 years [35].

Given this background of a high prevalence of interpersonal violence, particularly sexual violence experienced by reproductive aged and pregnant women, we conducted the present study to evaluate the reliability and validity (specifically the construct and criterion validity) of the Spanish-language version of the PCL-C against a Clinician-Administered PTSD Scale (CAPS) reference/gold standard among pregnant Peruvian women [36]. Additionally, we assessed the optimal cut-off points for discrimination between participants with and without PTSD using the PCL-C instrument. Evaluating the factor structure of a screening questionnaire when applied in new context or cultural background has been one of the widely-used methods of assessing psychometric properties. Hence, we also evaluated the factor structure of the PCL-C questionnaire using exploratory and confirmatory factor analytic approaches.

## Methods

### Study participants

This cross-sectional study was a part of the Pregnancy Outcomes, Maternal and Infant Study (PrOMIS) Cohort, which is an ongoing prospective cohort study. The study population was drawn from pregnant women receiving prenatal care at the Instituto Nacional Materno Perinatal (INMP) in Lima, Peru between February 2012 and March 2014. Under the aegis of the Peruvian Ministry of Health, the INMP is the primary referral health establishment for maternal and perinatal care. Pregnant women between the ages of 18 and 49 years, with a gestational age of ≤16 weeks, and who spoke and understood Spanish were eligible for inclusion.

### Study procedures

We used a two-stage study design where participants were first interviewed in-person by trained social workers using the PCL-C. Within 15 days of the initial interview, randomly selected participants were invited to take part in a diagnostic interview conducted by licensed psychologists who were blinded to the PCL-C questionnaire outcome using the CAPS. During the PCL-C interview, information pertaining to maternal sociodemographic, behavioral characteristics as well as medical and reproductive histories was ascertained. The questionnaire, originally written in English, was translated into Spanish by a team of native Spanish speakers (including co-author, SES). To ensure proper expression and conceptualization of terminologies in local contexts, the translated version was back-translated and modified until the back-translated version was comparable with the original English version.

#### Screening instrument

The PCL-C assesses posttraumatic stress disorder using 17 items designed according to the DSM– IV criteria. For each item, the participant is asked to indicate how much they have been bothered by each symptom over the past month on a 5-point Likert scale categorized as 1 (not at all), 2 (a little bit), 3 (moderately), 4 (quite a bit) and 5 (extremely) with regards to stressful life experiences. The PCL-C total score (i.e., sum of scores for the 17 items) ranges from 17 to 85. The PCL-C has been shown to have excellent internal consistency, test-retest reliability and convergent validity among both clinical and nonclinical populations [25–27, 37]. The Spanish-language version of the PCL-C has also been shown to have psychometric properties roughly similar to those reported for the English-language version [38]. An interviewer administered PCL-C has been used in prior studies (Additional file 1).

#### Diagnostic instrument-interview

The CAPS, the diagnostic criterion gold standard instrument selected for our study, was used to determine the presence of a PTSD diagnosis during the past 12 months. The CAPS is a structured interview for ascertainment of a PTSD diagnosis using DSM-IV criteria, including criterion A (exposure), criteria B – D (core symptoms clusters), criterion E (chronology), criterion F (functional impairment), and the associated symptoms of guilt and dissociation [36, 39]. The CAPS has excellent reliability, validity, diagnostic utility, and sensitivity to clinical change, and has been widely used as a standard criterion measure of PTSD [40].

#### Two-stage sample selection

Of the 3775 eligible participants, 3372 (89.3%) completed a structured in-person interview. Due to missing information on the PCL-C, 83 participants were excluded, leaving 3289 participants with completed PCL-C information in this analysis. Due to cost and time constraints, a subset of participants (*N* = 641, 19.5%) was randomly selected to complete a diagnostic interview within 15 days of the initial structured interview. Of the 641 women, eight participants missing information on PCL-C were excluded, and 633 participants with completed PCL-C and CAPS interview information remained in the diagnostic accuracy analysis.

### Statistical analysis

#### Reliability

We assessed the reliability of PCL-C using several agreement and consistency indices. Specifically, the Cronbach’s alpha (using the PCL-C total score) and item-total correlations were computed to assess the internal consistency and homogeneity of PCL-C items.

#### Criterion validity

The criterion validity for the PCL-C was assessed based on the CAPS diagnosis of PTSD. We computed the following operating characteristics: sensitivity, specificity, positive likelihood ratio, negative likelihood ratio, positive predictive values, and negative predictive values. To identify the best PCL-C cut-off score to use for PTSD screening of Spanish-speaking Peruvian pregnant women in early pregnancy, we completed receiver operating characteristic (ROC) curve analyses to identify optimal balance of sensitivity and specificity, area under the ROC curve (AUC) and we estimated the associated nonparametric 95% CI for the AUC. Additionally, we calculated the Youden Index, a metric used for identifying an optimum cut-off score for screening test. Briefly, the Youden index is a function of sensitivity and specificity calculated as the (sensitivity + specificity - 1). The range of the index is from 0 to 1 [41] with the higher values designating an optimum cut-off.

As noted above, because of cost and time constraints, we employed a two-stage design where a subset of participants screened with the PCL-C was assessed using the clinician administered CAPS. Since a subset of women screened with the PCL-C was selected for CAPS diagnostic interviews, it is possible to have verification bias. Given the likelihood of referral or verification bias [42], we implemented an analytical strategy that corrects for bias introduced as a result of our study design. Specifically, we evaluated the Begg and Greenes adjusted estimates of psychometric properties. Estimates generated from this analysis are corrected for verification bias using a Bayes Theorem approach [43] which applies a missing at random (MAR) assumption. Because it has been shown that the asymptotic confidence intervals might be unreliable in validation studies, we calculated bootstrapped confidence intervals to render estimated confidence intervals closer to their expected values [44].

#### Construct validity

Using exploratory factor analysis (EFA) and confirmatory factor analysis (CFA), we explored the factor structure of PCL-C. Prior to performing factor analysis, we assessed the suitability of the data for performing factor analysis. This analysis showed that it is appropriate to proceed with factor analysis (Bartlett’s test of sphericity (*p* < 0.001), and the Kaiser-Meyer-Olkin measure of sampling adequacy = 0.93). We then used the scree plot, presenting the eigenvalues associated with each factor, to identify the number of meaningful factors. Factors with relatively large eigenvalues (greater than one) were assumed to be meaningful and were retained for rotation. Factors with small eigenvalues were not retained [45]. Factor loadings >0.5 were used in the factor designation. To complement the EFA, we conducted CFA to evaluate the fit of three-factor and four-factor models identified in the literature corresponding to DSM-IV and DSM-5. The chi-square statistics, standardized root mean square residual (SRMR), the comparative fit index (CFI) and the root mean square error of approximation (RMSEA) were calculated. The following criteria are recommended to provide evidence for reasonably good fit [46]: 1) SRMR close to 0.08 or below; 2) CFI close to 0.95 or above; and 3) RMSEA close to 0.06 or below.

## Results

### Participant characteristics

A summary of selected sociodemographic and reproductive characteristics of study participants is presented in Table 1. A total of 3289 participants between the ages of 18 and 49 years (mean age = 28.2 years; standard deviation =6.3 years) participated in the study. The average gestational age at interview was 9.3 weeks (standard deviation =4.3 weeks). The majority of participants were Mestizos (75.3%), married or living with a partner (80.8%), and had at least 7 years of education (95.3%). About half of the participants were unemployed (53.9%), reported difficulty paying for the very basics (49.6%), nulliparous (48.9%) and reported the index pregnancy was unplanned (57.5%). The vast majority of participants reported experiencing physical or sexual abuse as a child [47] (70.2%), and one-third of participants reported experiencing lifetime physical or sexual intimate partner violence [48] (35.9%).

**Table 1:** Sociodemographic and reproductive characteristics of the study population

Characteristic	All		Diagnostic interview		PCL-C screening only		*P* value^a^
	(*N* = 3289)		(*N* = 633)		(*N* = 2656)		
	*n*	%	*n*	%	*n*	%	
Age (yr)^b^	28.2 ± 6.3		28.2 ± 6.1		28.2 ± 6.3		0.99
Age (yr)							
18–20	169	*5.1*	29	*4.6*	140	*5.3*	0.68
20–29	1836	*55.8*	367	*58.0*	1469	*55.3*	
30–34	692	*21.0*	128	*20.2*	564	*21.2*	
≥ 35	591	*18.0*	109	*17.2*	482	*18.1*	
Education (yr)							
≤ 6	143	*4.3*	25	*3.9*	118	*4.44*	0.75
7–12	1787	*54.3*	339	*53.6*	1448	*54.5*	
> 12	1349	*41.0*	267	*42.2*	1082	*40.7*	
Mestizo	2475	*75.3*	481	*76.0*	1994	*75.1*	0.65
Married/living with a partner	2658	*80.8*	518	*81.8*	2140	*80.6*	0.50
Employed	1515	*46.1*	307	*48.5*	1208	*45.5*	0.19
Access to basic food							
Hard	1630	*49.6*	302	*47.7*	1328	*50.0*	0.31
Not very hard	1657	*50.4*	331	*52.3*	1326	*49.9*	
Difficulty in paying for medical care							
Hard	1740	*52.9*	316	*49.9*	1424	*53.6*	0.11
Not very hard	1541	*46.9*	315	*49.8*	1226	*46.2*	
Nulliparous	1609	*48.9*	333	*52.6*	1276	*48.0*	0.04
Unplanned pregnancy	1892	*57.5*	352	*55.6*	1540	*58.0*	0.28
Gestational age at interview (wk)^b^	9.3 ± 4.3		9.6 ± 3.4		9.2 ± 4.7		0.02
Any lifetime sexual or physical abuse by intimate partner^c^							
No	2096	*63.7*	442	*69.8*	1654	*62.3*	<0.001
Yes	1181	*35.9*	188	*29.7*	993	*37.4*	
Any childhood physical or sexual abuse^d^							
No	937	*28.5*	190	*30.0*	747	*28.1*	0.38
Yes	2308	*70.2*	436	*68.9*	1872	*70.5*	

Due to missing data, percentages may not add up to 100%

^a^For continuous variables, *p* value was calculated using Student’s t test; for categorical variables, *p* value was calculated using either Chi-square test or Fisher’s exact test

^b^mean ± standard deviation

^c^Intimate partner abuse assessed using the Demographic Health Survey Questionnaires and Modules: Domestic Violence Module [48] and the WHO Multi-Country Study on Violence Against Women [68]

^d^Experiences of childhood physical and sexual abuse assessed using Childhood Physical and Sexual Abuse Questionnaire [47]

Twenty participants fulfilled the DSM-IV criteria for PTSD diagnosis based on CAPS interview (3% of the population). Overall PCL-C scores were higher among individuals with PTSD (median = 29; interquartile range: 27–35.25) compared to the non-PTSD individuals (median = 23; interquartile range: 19–28, *p* < 0.001) (Additional file 2).

### Reliability

The internal consistency of the PCL-C gave a Cronbach’s alpha of 0.90. The correlations between the 17 items of the PCL-C and the total scores ranged from 0.41 to 0.77 (all *p*-values <0.0001), indicating good relationship between each item and all the other items on the scale (Table 2).

**Table 2:** Item level values and item-total correlation of PCL-C

Item	Corrected item-total correlation	Alpha if Item deleted
1. Memories/thoughts/images	0.66	0.90
2. Repeated, disturbing dreams	0.62	0.90
3. Acting/feeling as if happening again	0.67	0.90
4. Feeling upset at reminders	0.77	0.89
5. Physical reactions at reminders	0.67	0.90
6. Avoid thinking/talking/feelings	0.72	0.89
7. Avoid activities/situations	0.73	0.89
8. Trouble remember details	0.59	0.90
9. Loss of interest in activities	0.60	0.90
10. Feeling distant or cut off from others	0.63	0.90
11. Feeling emotionally numb	0.56	0.90
12. Feeling as if future cut short	0.61	0.90
13. Trouble sleeping	0.51	0.90
14. Irritability/angry outbursts	0.66	0.90
15. Difficulty concentrating	0.61	0.90
16. Super alert or watchful	0.41	0.90
17. Jumpy or easily started	0.63	0.90
Sum score	N/A	0.90^a^

^a^Overall Cronbach’s alpha based on sum score; *PCL-C* Posttraumatic Stress Disorders Checklist Civilian Version

### Validity

#### Criterion validity

Using the CAPS as the criterion gold standard, the optimal cut-off of PCL-C for maximizing the sensitivity without loss of specificity was a score of ≥26 (Table 3). At the optimal cut-off score, the sensitivity and specificity of PCL-C for detecting PTSD in this population were 0.86 (95% CI: 0.78–0.92) and 0.63 (95% CI: 0.62–0.65), respectively. The positive likelihood ratio was 2.35 (95% CI: 2.16–2.57) with negative likelihood ratio of 0.22 (95% CI: 0.14–0.35). This suggested that women with PTSD were 2.35 times more likely than women without PTSD to have a PCL-C score equal or greater than 26, and were 0.22 times as likely to have a PCL-C score less than 26. The positive predicted value was 0.08 (95% CI: 0.06–0.09) and the negative predicted value was 0.99 (95% CI: 0.99–1.00). The area under the ROC curve for detecting PTSD at a PCL-C score of 26 or higher was 0.75 (95% CI: 0.71–0.78) (*P* < 0.01) (Additional file 3). The results largely remained similar when stratified by experiences of lifetime sexual or physical abuse by an intimate partner (Additional files 4 and 5).

**Table 3:** Sensitivity and specificity for PTSD diagnosis across various cut-off scores of PCL-C

Cut-off Score	Sensitivity (95% CI)	Specificity (95% CI)	Youden index	+PV (95% CI)	-PV (95% CI)	+LR (95% CI)	-LR (95% CI)	PR
20	0.95 (0.90, 0.99)	0.23 (0.22, 0.25)	0.18	0.04 (0.03, 0.05)	0.99 (0.98, 1.00)	1.24 (1.19, 1.30)	0.20 (0.08, 0.47)	0.77
22	0.91 (0.84, 0.96)	0.41 (0.39, 0.43)	0.32	0.05 (0.04, 0.06)	0.99 (0.99, 1.00)	1.53 (1.44, 1.64)	0.22 (0.12, 0.41)	0.60
24	0.86 (0.78, 0.92)	0.54 (0.52, 0.55)	0.40	0.06 (0.05, 0.07)	0.99 (0.99, 1.00)	1.86 (1.70, 2.02)	0.26 (0.16, 0.42)	0.48
**26**	**0.86 (0.78, 0.92)**	**0.63 (0.62, 0.65)**	**0.49**	**0.08 (0.06, 0.09)**	**0.99 (0.99, 1.00)**	**2.35 (2.16, 2.57)**	**0.22 (0.14, 0.35)**	**0.38**
28	0.68 (0.58, 0.77)	0.70 (0.69, 0.72)	0.38	0.07 (0.06, 0.09)	0.99 (0.98, 0.99)	2.29 (1.99, 2.64)	0.46 (0.35, 0.60)	0.31
30	0.49 (0.39, 0.59)	0.76 (0.74, 0.77)	0.25	0.06 (0.05, 0.08)	0.98 (0.97, 0.98)	2.01 (1.64, 2.46)	0.67 (0.56, 0.81)	0.25
35	0.40 (0.31, 0.50)	0.87 (0.86, 0.88)	0.27	0.09 (0.07, 0.12)	0.98 (0.97, 0.98)	3.01 (2.35, 3.84)	0.69 (0.59, 0.81)	0.14
40	0.26 (0.18, 0.36)	0.91 (0.90, 0.92)	0.17	0.10 (0.06, 0.13)	0.97 (0.97, 0.98)	3.05 (2.19, 4.25)	0.81 (0.72, 0.90)	0.09
45	0.13 (0.07, 0.21)	0.95 (0.94, 0.95)	0.08	0.08 (0.04, 0.12)	0.97 (0.96, 0.98)	2.46 (1.48, 4.09)	0.92 (0.85, 0.99)	0.06

*Abbreviations*: *+PV* positive predicted value, *−PV* negative predicted value, *+LR* positive likelihood ratio, *−LR* negative likelihood ratio, *PR* prevalence; Bold text indicates the optimum cut-off

#### Construct validity

The results obtained from EFA indicated a three-factor solution (Table 4) with eigenvalues greater than 1.0, which accounted for 55% of the common variance. The factor loadings of all 17 items were greater than 0.5. Factor 1 consisted primarily of re-experiencing and avoidance symptoms, factor 2 consisted of dysphoria symptoms, and factor 3 was primarily comprised of hyperarousal symptoms.

**Table 4:** Exploratory factor analysis of PCL-C

Item	Factor 1	Factor 2	Factor 3
1. Memories/thoughts/images	**0.68**	0.07	0.01
2. Repeated, disturbing dreams	**0.70**	0.11	−0.15
3. Acting/feeling as if happening again	**0.74**	0.10	−0.11
4. Feeling upset at reminders	**0.74**	0.04	0.16
5. Physical reactions at reminders	**0.65**	0.07	0.07
6. Avoid thinking/talking/feelings	**0.74**	−0.11	0.28
7. Avoid activities/situations	**0.75**	−0.08	0.25
8. Trouble remember details	**0.59**	0.19	−0.14
9. Loss of interest in activities	0.15	**0.60**	-0.05
10. Feeling distant or cut off from others	0.10	**0.69**	-0.03
11. Feeling emotionally numb	0.10	**0.70**	-0.15
12. Feeling as if future cut short	0.13	**0.65**	-0.04
13. Trouble sleeping	−0.11	**0.54**	0.27
14. Irritability/angry outbursts	−0.04	0.49	**0.53**
15. Difficulty concentrating	0.00	**0.60**	0.20
16. Super alert or watchful	0.07	−0.17	**0.78**
17. Jumpy or easily started	−0.05	0.44	**0.55**

Factor loadings >0.5 are in bold

**Fig. 1:**
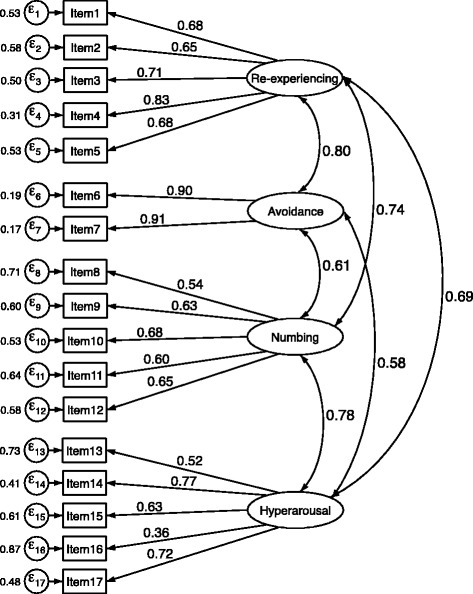
Path diagram of the selected confirmatory factor analysis model

Additional file 6 details the item mapping for the model from EFA analysis (Model 1), models identified from previous studies (Model 2–4) that underwent CFA. In addition to our EFA, we carried out a CFA to examine the construct validity and to test whether the factorial structure of PCL-C has a three-factor model. The three-factor model (Model 2) based on DSM-IV symptom structure had reasonable fit statistics with comparative fit index of 0.86 and RMSEA of 0.09). Model 3, a four-factor mode, corresponding to DSM-5, had better-fit statistics with CFI of 0.94. The RMSEA (0.06) and SRMR (0.04) values were acceptable indicating reasonable error of approximation (Table 5). Detailed path diagram, factor loadings as well as item variances are shown in Fig. 1 corresponding to DSM-5 factors of re-experiencing, avoidance, numbing and hyperarousal.

**Table 5:** Fit indices for confirmatory factor analysis models

Model	Chi-sq. (df)	SRMR	RMSEA	CFI
1	2344.22 (116)	0.04	0.08	0.90
2	3379.95 (116)	0.07	0.09	0.86
**3**	**1404.67 (113)**	**0.04**	**0.06**	**0.94**
4	1830.05 (113)	0.05	0.07	0.93

Abbreviations: *Chi-sq. (df)* chi-square statistic (degree of freedom), *SRMR* standardized root mean square residual, *RMSEA* root mean square error of approximation, *CFI* comparative fit index; Bold indicates the best fitting model

Model 1: Factor 1—1,2,3,4,5,6,7,8; Factor 9,10,11,12,13,15; Factor 3—14,16,17

Model 2: Factor 1—1,2,3,4,5,6,7,8; Factor 2—9,10,11,12,13,15; and Factor 3—14,17

Model 3: Factor 1—1,2,3,4,5; Factor 2—6,7; Factor 3—8,9,10,11,12; and Factor 4—13,14,15,16,17

Model 4: Factor 1—1,2,3,4,5; Factor 2—6,7; Factor 3—8,9,10,11,12,13,14,15 and Factor 4—16,17

## Discussion

To our knowledge, this is the first validation study of the Spanish-language version of the PCL-C questionnaire as a screening tool for PTSD in pregnancy. When compared with a criterion gold standard (CAPS), the PCL-C has a good validity (sensitivity (86%) and specificity (63%) for detecting PTSD symptoms among Peruvian pregnant women. Our ROC analysis and Youden index showed that a threshold of 26 on the PCL-C was the most appropriate cut-off and offered the optimal discriminatory power in detecting PTSD in our study population. In addition, our study provided strong evidence for the construct validity of the PCL-C with a four-factor structure consisting of re-experiencing, avoidance, numbing and hyperarousal. Our findings are consistent with reports from prior studies [49–53].

In our study, the sensitivity and specificity of PCL-C at the optimal cut-off score of 26 or greater was 0.86 (95% CI: 0.78–0.92) and 0.63 (95% CI: 0.62–0.65), respectively. An excellent negative predicted value of 0.99 was obtained at the optimal cut-off point of 26. The positive predicted value (PPV) was relatively poor (0.08), partly due to the low prevalence (3%) of PTSD in the population. The PPV depends on sensitivity, specificity, and prevalence of PTSD among study participants. In our population, the relatively low 12-month prevalence of PTSD (3%) based on the CAPS diagnosis likely accounts for the low PPV in our study. In the original validation study of PCL-C conducted among motor vehicle accident and sexual assault victims Blanchard et al. found the optimal cut-off score of 44 resulted in a sensitivity of 94% and specificity of 86% when compared with CAPS reference standard [54]. Subsequently, other investigators have evaluated the validity of PCL-C reporting optimum cut-off scores ranging from 28 to 60 (Additional file 1). McDonald and Calhoun have reported similar observations in their review of 12 studies that used PCL-C [55]. The cut-off scores in our study were similar with some studies. For instance, Walker et al. in their study of women attending a health maintenance organization (HMO) in Seattle found that the optimum PCL-C cut-off score was 30 with a sensitivity of 82% and specificity of 76% [56]. Andrykowski et al. in their study of women with breast cancer found the optimum PCL-C cut-off score to be 30 yielding a sensitivity of 100% and specificity of 83% [57]. The wide variation in cut-off scores between studies may be attributable to differences in prevalence of PTSD, demographics, disease severity, comorbidity, and other characteristics of populations. We do not have a clear explanation for the low prevalence of PTSD by CAPS in our study. One potential reason is that high levels of history of childhood abuse, intimate partner violence, anxiety about pregnancy, delivery and health of the fetus may have desensitized them to PTSD symptoms or make them feel less comfortable discussing the PTSD symptoms in a face-to-face interview with the psychologists. Future studies are needed to provide further insights into this issue. Moreover, our study was conducted in early pregnancy. It is possible that vulnerability to PTSD symptoms might increase throughout pregnancy due to physiological and neuroendocrine changes associated with pregnancy, fear of childbirth & pain, and ongoing exposure to violence as the pregnancy progresses [13].

Using exploratory and confirmatory factor analytic approaches, we confirmed the multi-dimensionality of the PCL-C, which is consistent with the majority of studies conducted in primary care settings [52, 53, 58] or general population-based research setting [59]. [60] The four-factor model proposed by King et al. [61] with four inter-correlated factors (re-experiencing, avoidance, numbing and hyperarousal) provided the best fit. Although the PCL-C items map directly onto criteria B (re-experiencing), C (avoidance/numbing) and D (hyperarousal) of DSM-IV PTSD diagnosis, the three-factor model did not provide the best fit in the present study. These findings are consistent with results from previous studies that found a four-factor model of PCL-C using either exploratory or confirmatory factor analysis [52, 58, 60–63].

Of note, several investigators have questioned the three-factor model of PCL-C conceptualizing DSM-IV criteria as little empirical data supports this hypothesis [23]. The current definition PTSD according to DSM-5 also suggests that avoidance and numbing may be two conceptually independent factors and should be split into avoidance cluster and negative alterations in cognition and mood cluster [64, 65]. In sum, the results of our study and those of others [60, 66, 67] indicate that a four-factor model of PCL-C may best represent the latent structure of PTSD [26].

Overall, the PCL-C demonstrated good item-total correlations. Compared to other items, Item 16 (Being “super alert” or watchful on guard) had the lowest item-total correlation (0.41) and loaded only moderately on the factor. Other investigators have reported similar lower item-total correlation and lower loading of Item 16 [32, 52, 66, 67]. It is not clear whether the low loading was due to wording property, a subtype of PTSD or potential confounding associated with pregnancy and life condition. Future exploration is required to empirically test the subtype hypothesis and validate the PCL-C, especially for the Spanish-language version across regions and populations in other Spanish-speaking countries.

Our study has several strengths including the use of a diagnostic gold standard to assess validity, a relatively large sample size, and execution of a rigorous analytic plan. Furthermore, our study expands the literature by including assessment of the Spanish-language version of the PCL-C among pregnant women. Despite these strengths, some limitations much be considered when interpreting our findings. First, four psychologists conducted the diagnostic interviews, and their inter-rater reliability was not calculated. Second, as anxiety levels might vary during the course of pregnancy, longitudinal studies are warranted to help understand how PTSD symptom severity changes across pregnancy trimesters. Finally, test-retest reliability was not calculated in our study.

## Conclusion

In conclusion, our results suggest that the Spanish-language version of the PCL-C may be used as a screening tool for pregnant women. The PCL-C has good reliability, factorial validity, and concurrent validity. In practical use, the optimal cut-off score in this study (PCL-C score ≥ 26) obtained by maximizing the overall rate of correct classification should be considered cautiously; woman who screened positive may require further investigation to confirm PTSD diagnosis. The patient’s pre-test probability of PTSD as well as the different costs of misclassification errors need to be taken into account for choosing an optimal cut-off score that minimize the average loss in classifying patients [42]. Further studies that evaluate the utility of correct classification would provide more evidence for determining the optimal cut-off score for pregnant women.

## Additional files

Additional file 1: Posttraumatic Stress Disorders Checklist Civilian Version (PCL-C) cut-off score used in previous studies. (DOCX 35 kb)

Additional file 2: Distribution of PCL-C scores according to clinician diagnosed PTSD status. (DOCX 20 kb)

Additional file 3: Receiver Operating Characteristic (ROC) Curves of PCL-C Score. (DOCX 23 kb)

Additional file 4: Sensitivity and specificity for PTSD diagnosis across various cut-off scores of Posttraumatic Stress Disorders Checklist Civilian Version (PCL-C) among subjects with lifetime sexual or physical abuse by intimate partner. (DOCX 17 kb)

Additional file 5: Sensitivity and specificity for PTSD diagnosis across various cut-off scores of Posttraumatic Stress Disorders Checklist Civilian Version (PCL-C) among subjects without lifetime sexual or physical abuse by intimate partner. (DOCX 17 kb)

Additional file 6 Item mapping for confirmatory factor analysis. (DOCX 16 kb)

## References

[1] APA (2013). Diagnostic and statistical manual of mental disorders (5th ed.).

[2] APA (1994). Diagnostic and statistical manual of mental disorders (4th ed.).

[3] Kessler RC, Sonnega A, Bromet E, Hughes M, Nelson CB (1995). Posttraumatic stress disorder in the national comorbidity survey. Arch Gen Psychiatry.

[4] Van Ameringen M, Mancini C, Patterson B, Boyle MH (2008). Post-traumatic stress disorder in Canada. CNS Neurosci Ther.

[5] Dorrington S, Zavos H, Ball H, McGuffin P, Rijsdijk F, Siribaddana S, Sumathipala A, Hotopf M (2014). Trauma, post-traumatic stress disorder and psychiatric disorders in a middle-income setting: prevalence and comorbidity. Br J Psychiatry J Ment Sci.

[6] Sareen J (2014). Posttraumatic stress disorder in adults: impact, comorbidity, risk factors, and treatment. La Revue canadienne de psychiatrie.

[7] Seng JS, Low LK, Sperlich M, Ronis DL, Liberzon I (2009). Prevalence, trauma history, and risk for posttraumatic stress disorder among nulliparous women in maternity care. Obstet Gynecol.

[8] Breslau N (2001). The epidemiology of posttraumatic stress disorder: what is the extent of the problem?. J Clin Psychiatry.

[9] Breslau N, Davis GC, Peterson EL, Schultz L (1997). Psychiatric sequelae of posttraumatic stress disorder in women. Arch Gen Psychiatry.

[10] Onoye JM, Shafer LA, Goebert DA, Morland LA, Matsu CR, Hamagami F (2013). Changes in PTSD symptomatology and mental health during pregnancy and postpartum. Arch Womens Ment Health.

[11] Rodriguez MA, Heilemann MV, Fielder E, Ang A, Nevarez F, Mangione CM (2008). Intimate partner violence, depression, and PTSD among pregnant Latina women. Ann Fam Med.

[12] Ross LE, McLean LM (2006). Anxiety disorders during pregnancy and the postpartum period: a systematic review. J Clin Psychiatry.

[13] Seng JS, Rauch SA, Resnick H, Reed CD, King A, Low LK, McPherson M, Muzik M, Abelson J, Liberzon I (2010). Exploring posttraumatic stress disorder symptom profile among pregnant women. J Psychosom Obstet Gynaecol.

[14] Seng JS, Oakley DJ, Sampselle CM, Killon C, Graham-Bermann S, Liberzon I (2001). Posttraumatic stress disorder and pregnancy complications. Obstet Gynecol.

[15] Shaw JG, Asch SM, Kimerling R, Frayne SM, Shaw KA, Phibbs CS (2014). Posttraumatic stress disorder and risk of spontaneous preterm birth. Obstet Gynecol.

[16] Lipkind HS, Curry AE, Huynh M, Thorpe LE, Matte T (2010). Birth outcomes among offspring of women exposed to the September 11, 2001, terrorist attacks. Obstet Gynecol.

[17] Yonkers KA, Smith MV, Forray A, Epperson CN, Costello D, Lin H, Belanger K (2014). Pregnant women with posttraumatic stress disorder and risk of preterm birth. JAMA Psychiatry.

[18] Jacob KS, Sharan P, Mirza I, Garrido-Cumbrera M, Seedat S, Mari JJ, Sreenivas V, Saxena S (2007). Mental health systems in countries: where are we now?. Lancet.

[19] Meltzer EC, Averbuch T, Samet JH, Saitz R, Jabbar K, Lloyd-Travaglini C, Liebschutz JM (2012). Discrepancy in Diagnosis and Treatment of Post-traumatic Stress Disorder (PTSD): Treatment for the Wrong Reason. J Behav Health Serv Res.

[20] Mahenge B, Stockl H, Likindikoki S, Kaaya S, Mbwambo J. The prevalence of mental health morbidity and its associated factors among women attending a prenatal clinic in Tanzania. Int J Gynaecol Obstet. 2015. 10.1016/j.ijgo.2015.04.032 26094728

[21] Neuner F, Elbert T (2007). The mental health disaster in conflict settings: can scientific research help?. BMC Public Health.

[22] Salyers MP, Evans LJ, Bond GR, Meyer PS (2004). Barriers to assessment and treatment of posttraumatic stress disorder and other trauma-related problems in people with severe mental illness: clinician perspectives. Community Ment Health J.

[23] Elhai JD, Palmieri PA (2011). The factor structure of posttraumatic stress disorder: a literature update, critique of methodology, and agenda for future research. J Anxiety Disord.

[24] Weathers FW, Huska JA, Keane TM. PCL-C for DSM-IV. In*.* Edited by Division NCfPBS. Boston; 1991.

[25] Weathers F, Litz B, Herman D, Huska J, Keane T: The PTSD Checklist (PCL): Reliability, Validity, and Diagnostic Utility. In: The Annual Convention of the International Society for Traumatic Stress Studies. San Antonio, TX; 1993.

[26] Wilkins KC, Lang AJ, Norman SB (2011). Synthesis of the psychometric properties of the PTSD checklist (PCL) military, civilian, and specific versions. Depress Anxiety.

[27] Conybeare D, Behar E, Solomon A, Newman MG, Borkovec TD (2012). The PTSD checklist-civilian version: reliability, validity, and factor structure in a nonclinical sample. J Clin Psychol.

[28] Blevins CA, Weathers FW, Davis MT, Witte TK, Domino JL (2015). The posttraumatic stress disorder checklist for DSM-5 (PCL-5): development and initial psychometric evaluation. J Trauma Stress.

[29] Vera-Villarroel P, Zych I, Celis-Atenas K, Cordova-Rubio N, Buela-Casal G (2011). Chilean validation of the Posttraumatic Stress Disorder Checklist-Civilian version (PCL-C) after the earthquake on February 27, 2010. Psychol Rep.

[30] Costa MF, Mendlowicz MV, Vasconcelos AG, Berger W, Luz MP, Figueira I, Garcia Rosa ML (2011). Confirmatory factor analysis of posttraumatic stress symptoms in Brazilian primary care patients: an examination of seven alternative models. J Anxiety Disord.

[31] Lima EP, Barreto SM, Assunção AA (2012). Factor structure, internal consistency and reliability of the posttraumatic Stress Disorder Checklist (PCL): an exploratory study. Trends Psychiatry Psychother.

[32] Passos RBF, Figueira I, Mendlowicz MV, Moraes CL, Coutinho ESF (2012). Exploratory factor analysis of the Brazilian version of the post-traumatic stress disorder checklist - civilian version (PCL-C). Rev Bras Psiquiatr.

[33] Ellsberg M, Jansen HA, Heise L, Watts CH, Garcia-Moreno C, Health WHOM-cSoWs, Domestic Violence against Women Study T (2008). Intimate partner violence and women’s physical and mental health in the WHO multi-country study on women’s health and domestic violence: an observational study. Lancet.

[34] Perales MT, Cripe SM, Lam N, Sanchez SE, Sanchez E, Williams MA (2009). Prevalence, types, and pattern of intimate partner violence among pregnant women in Lima, Peru. Violence Against Women.

[35] Barrios YV, Gelaye B, Zhong Q, Nicolaidis C, Rondon MB, Garcia PJ, Sanchez PA, Sanchez SE, Williams MA (2015). Association of childhood physical and sexual abuse with intimate partner violence, poor general health and depressive symptoms among pregnant women. PLoS One.

[36] Blake D, Weathers F, Nagy L, Kaloupek D, Klauminzer G, Charney D, Keane T, Buckley TC, Disorder NCPS (2000). Instruction manual clinician-administered PTSD Scale (CAPS).

[37] Mueser KT, Rosenberg SD, Fox L, Salyers MP, Ford JD, Carty P (2001). Psychometric evaluation of trauma and posttraumatic stress disorder assessments in persons with severe mental illness. Psychol Assess.

[38] Marshall GN (2004). Posttraumatic stress disorder symptom checklist: factor structure and english–spanish measurement invariance. J Trauma Stress.

[39] Weathers FW, Keane TM, Davidson JR (2001). Clinician-administered PTSD scale: a review of the first ten years of research. Depress Anxiety.

[40] Weathers FW, Keane TM, Davidson JRT (2001). Clinician-administered PTSD scale: a review of the first ten years of research. Depress Anxiety.

[41] Fluss R, Faraggi D, Reiser B (2005). Estimation of the Youden index and its associated cutoff point. Biom J.

[42] Zhou XH, Obuchowski NA, Mcclish DK (2011). Statistical methods in diagnostic medicine.

[43] Begg CB, Greenes RA (1983). Assessment of diagnostic tests when disease verification is subject to selection bias. Biometics.

[44] Carpenter J, Bithell J (2000). Bootstrap confidence intervals: when, which, what? A practical guide for medical statisticians. Stat Med.

[45] Kaiser HF (1960). The application of electronic computers to factor analysis. Educ Psychol Meas.

[46] Brown TA (2006). Confirmatory factor analysis for applied research.

[47] Felitti VJ, Anda RF, Nordenberg D, Williamson DF, Spitz AM, Edwards V, Koss MP, Marks JS (1998). Relationship of childhood abuse and household dysfunction to many of the leading causes of death in adults. The Adverse Childhood Experiences (ACE) Study. Am J Prev Med.

[48] DHS: Demographic Health Survey questionnaires and modules: domestic violence module. Available at: http://www.measuredhs.com/aboutsurveys/dhs/modules_archive.cfm. 2005. Accessed 19 Sept 2014.

[49] DeVellis RF (2003). Scale development: theory and applications.

[50] Keen SM (2008). Psychometric properties of PTSD Checklist in sample of male veterans. J Rehabil Res Dev.

[51] Bollinger A, Cuevas C, Vielhauer M, Morgan E, Keane T (2008). The operating characteristics of the PTSD checklist in detecting PTSD in HIV+ substance abusers. J Psychol Trauma.

[52] Cuevas C, Bollinger A, Vielhauer M, Morgan E, Sohler N, Brief D, Miller A, Keane T (2007). HIV/AIDS cost study: construct validity and factor structure of the PTSD checklist in dually diagnosed HIV-seropositive adults. Journal of Trauma Practice.

[53] Cook JM, Elhai JD, Arean PA (2005). Psychometric properties of the PTSD Checklist with older primary care patients. J Trauma Stress.

[54] Blanchard EB, Jones-Alexander J, Buckley TC, Forneris CA (1996). Psychometric Properties of the PTSD checklist (PCL). Behav Res Ther.

[55] McDonald SD, Calhoun PS (2010). The diagnostic accuracy of the PTSD checklist: a critical review. Clin Psychol Rev.

[56] Walker EA, Newman E, Dobie DJ, Ciechanowski P, Katon W (2002). Validation of the PTSD checklist in an HMO sample of women. Gen Hosp Psychiatry.

[57] Andrykowski MA, Cordova MJ, Studts JL, Miller TW (1998). Posttraurnatic Stress D isorder after treatment for breast cancer: prevalence of diagnosis and use of the PTSD checklist—civilian version (PCL-C) as a screening instrument. J Consult Clin Psychol.

[58] DuHamel KN, Ostrof J, Ashman T, Winkel G, Mundy EA, Keane TM, Morasco BJ, Vickberg SM, Hurley K, Burkhalter J (2004). Construct validity of the posttraumatic stress disorder checklist in cancer survivors: analyses based on two samples. Psychol Assess.

[59] Vera-Villarroel P, Zych I, Celis-Atenas K, Cordova-Rubio N, Buela-Casal G (2011). Chilean validation of the posttraumatic stress disorder checklist-civilian version (PCL-C) after the earthquake on February 27, 2010. Psychol Rep.

[60] Schinka JA, Brown LM, Borenstein AR, Mortimer JA (2007). Confirmatory factor analysis of the PTSD checklist in the elderly. J Tramatic Stress.

[61] King DW, Leskin GA, King LA, Weathers FW (1998). confirmatory factor analysis of the clinician-administered PTSD scale: evidence for the dimensionality of posttraumatic stress disorder. Psychol Assess.

[62] Asmundson GJ, Wright KD, McCreary DR, Pedlar D (2003). Post-traumatic stress disorder symptoms in United Nations peacekeepers: an examination of factor structure in peacekeepers with and without chronic pain. Cogn Behav Ther.

[63] Palmieri PA, Weathers FW, Difede J, King DW (2007). Confirmatory factor analysis of the PTSD checklist and the clinician-administered PTSD Scale in disaster workers exposed to the world trade center ground zero. J Abnorm Psychol.

[64] Foa EB, Zinbarg R, Rothbaum BO (1992). Uncontrollability and unpredictability in post-traumaticstress disorder: an animalmodel. Psychol Bull.

[65] Taylor S, Kuch K, Koch WJ, Crockett DJ, Passey G (1998). The structure of posttraumatic stress symptoms. J Abnorm Psychol.

[66] Shelby RA, Golden-Kreutz DM, Andersen BL (2005). Mismatch of posttraumatic stress disorder (PTSD) symptoms and DSM-IV symptom clusters in a cancer sample: exploratory factor analysis of the PTSD checklist-civilian version. J Trauma Stress.

[67] Smith MY, Redd W, DuHamel K, Vickberg SJ, Ricketts P (1999). Validation of the PTSD checklist-civilian version in survivors of bone marrow transplantation. J Trauma Stress.

[68] Garcia-Moreno C, Jansen HA, Ellsberg M, Heise L, Watts CH (2006). Prevalence of intimate partner violence: findings from the WHO multi-country study on women’s health and domestic violence. Lancet.

