# Supplementary material for: Validity of the posttraumatic stress disorders (PTSD) checklist in pregnant women
Source: BMC Psychiatry. 2017 May 12;17:179. doi: 10.1186/s12888-017-1304-4 (PMC5427611; doi:10.1186/s12888-017-1304-4)
Supplement: Supplementary file 1 — Posttraumatic Stress Disorders Checklist Civilian Version (PCL-C) cut-off score used in previous studies. (DOCX 35 kb) [file 12888_2017_1304_MOESM1_ESM.docx]

**Supplemental Table 2. Posttraumatic Stress Disorders Checklist Civilian Version (PCL-C) Cut-off Score Used in Previous Studies**

| Author | Study population | Gold standard | PR | Optimal cut-off score | Sensitivity | Specificity | AUC | Assessment | Diagnostic efficiency | Study findings |
| --- | --- | --- | --- | --- | --- | --- | --- | --- | --- | --- |
| Blanchard [1] | 40 adults who were victims of severe automobile accident (n=27) or a sexual assault (n=13) | CAPS | 0.45 | 44 | 0.944 | 0.764 | - | SR | 0.90 | Prespecified cut-off points of 50 and 44 were compared. |
| Andrykowski [2] | 72 women posttreatment for breast cancer | SCID | 0.06 | 30 | 1.00 | 0.83 | - | INT | 0.80 | Prespecified cut-off points of 30, 35, 40, 45 and 50 were compared. |
| Manne [3] | 65 mothers of pediatric cancer survivors | SCID | 0.06 | 40 | 1.00 | 0.77 | - | SR | 0.79 | Prespecified cut-off points of 40, 45 and 50 were compared. |
| Dobie [4] | 282 women seen for care at the Veterans Affairs (VA) Puget Sound Healthcare system | CAPS | 0.36 | 38 | 0.79 | 0.79 | 0.86 | SR | - | Prespecified cut-off points of 30, 44, 50, 60 and the optimal cut-off score (38) were compared. |
| Walker [5] | 261 female HMO members | CAPS | 0.11 | 30 | 0.82 | 0.76 | 0.84 | SR | - | Prespecified cut-off points of 25, 30, 35, 40, 45, 50 and 55 were compared. |
| Lang[6] | 49 women seen in the VA San Diego Healthcare System | CIDI | 0.31 | 28 | 0.94 | 0.68 | 0.89 | SR | 0.78 | Cut-off points in the range of 26 to 40 and 50 were compared. |
| Ruggiero [7] | 392 college students | CAPS | - | 44 | 0.90 | 0.95 | - | SR | 0.95 | Prespecified cut-off points of 50 and 44 were compared. |
| Cook [8] | 142 older adult primary care patients | CAPS | - | 37 | 0.96 | 0.92 | 0.98 | INT |  | Cut-off points in the range of 32 to 42 were compared. |
| Grubaugh [9] | 44 traumatized, adult, public-sector mental health patients | CAPS | 0.59 | 54 | 0.69 | 0.78 | 0.76 | INT |  | All scores of the participants in the study were used as cut-off points for comparison |
| Harrington [10] | 44 women substance users | CAPS | 0.39 | 44 | 0.76 | 0.79 | - | SR | 0.78 | Cut-off points in the range of 35 to 41, 44 and 46 were compared. |
| Bollinger [11] | 57 HIV-seropositive patients | CAPS | 0.12 | 52 | 0.86 | 0.79 | 0.91 | SR | 0.82 | Results using cut-off score of 50 and 52 were provided. |
| Hudson [12] | 100 patients being treated for medical and/or psychiatric conditions | CAPS | 0.10 | 36 | 0.90 | 0.87 | 0.935 | SR |  | Optimum cut-off point was indicated from ROC analysis. |
| Keen [13] | 114 male veterans | CAPS | 0.22 | 60 | 0.56 | 0.92 | 0.86 | SR | - | Cut-off points in the range of 43 to 71 were compared. |
| Freedy[14] | 411 Family Practice Center patients | CAPS | 0.32 | 43 | 0.80 | 0.81 | 0.93 | INT | - | Cut-off points in the range of 37 to 49 were compared. |
| Chiu [15] | 1915 retired firefighters exposed to the World trade Center disaster | DIS | 0.06 | 39 | 0.85 | 0.82 | 0.91 | SR | - | Cut-off points in the range of 27 to 50 were compared. |
| Pietrzak [16]^a^ | 206 older adults who experienced Hurricane Ike | PCL-based DSM-IV diagnosis | 0.07 | 39 | 0.96 | 0.92 | 0.98 | INT | 0.92 | Cut-off points in the range of 34 to 50 were compared. |
|  |  |  | 0.08 | 37 | 1.00 | 0.90 | 0.98 | INT | 0.91 |  |
| Gardner [17] | 132 outpatients with burns | DSM-IV diagnosis | 0.39 | 50 | 0.90 | 0.79 | - | SR | 0.83 | Pre-specified cut-off points of 45, 50 and 55 were compared. |
| Karstoft [18] | 415 Danish soldiers | SIDI | 0.07 | 40 | 0.79 | 0.93 | 0.95 | SR | - | Cut-off points in the range of 36 to 54 were compared. |

Abbreviations: CAPS, Clinician-Administered PTSD Scale; CIDI, Composite International Diagnostic Interview; SCID, Structured Clinical Interview for DSM; Disorder; DIS: Diagnostic Interview Schedule; AUC, area under the Receiver Operating Characteristics curves; PR, prevalence

^a^ The first row of this study refers to worst event-related PTSD, while the second row refers to Hurricane Ike-related PTSD; SR, self-report; INT, interview

**References** **Supplemental Table 2**

1. Blanchard EB, Jones-Alexander J, Buckley TC, Forneris CA: **Psychometric Properties of the PTSD checklist (PCL)**. *Behaviour Research and Therapy* 1996, **34**(8):669-673.

2. Andrykowski MA, Cordova MJ, Studts JL, Miller TW: **Posttraurnatic Stress D isorder After Treatment for Breast Cancer: Prevalence of Diagnosis and Use of the PTSD Checklist—Civilian Version (PCL-C) as a Screening Instrument**. *Journal of Consulting and ClinicalPsychology* 1998, **66**(3):586-590.

3. Manne SL, Hamel KD, Gallelli K, Sorgen K, Redd WH: **Posttraumatic Stress Disorder Among Mothers of Pediatric Cancer Survivors: Diagnosis, Comorbidity, and Utility of the PTSD Checklist as a Screening Instrument**. *Journal of Pediatric Psychology* 1998, **23**(6):357-366.

4. Dobie DJ, Kivlahan DR, Maynard C, Bush KR, McFall M, Epler AJ, Bradley KA: **Screening for post-traumatic stress disorder in female Veteran’s Affairs patients: validation of the PTSD checklist**. *General Hospital Psychiatry* 2002, **24**:367-374.

5. Walker EA, Newman E, Dobie DJ, Ciechanowski P, Katon W: **Validation of the PTSD checklist in an HMO sample of women**. *General Hospital Psychiatry* 2002, **24**:375-380.

6. Lang AJ, Laffaye C, Satz LE, Dresselhaus TR, Stein MB: **Sensitivity and Specificity of the PTSD Checklist in Detecting PTSD in Female Veterans in Primary Care**. *Journal of Traumatic Stress* 2003, **16**(3):257-264.

7. Ruggiero KJ, Ben KD, Scotti JR, Rabalais AE: **Psychometric Properties of the PTSD Checklist—Civilian Version**. *Journal of Traumatic Stress* 2003, **16**(5):495-502.

8. Cook JM, Elhai JD, Arean PA: **Psychometric properties of the PTSD Checklist with older primary care patients**. *Journal of Traumatic Stress* 2005, **18**(4):371-376.

9. Grubaugh AL, Elhai JD, Cusack KJ, Wells C, Frueh BC: **Screening for PTSD in public-sector mental health settings: the diagnostic utility of the PTSD checklist**. *Depression and anxiety* 2007, **24**(2):124-129.

10. Harrington T, Newman E: **The psychometric utility of two self-report measures of PTSD among women substance users**. *Addictive behaviors* 2007, **32**(12):2788-2798.

11. Bollinger A, Cuevas C, Vielhauer M, Morgan E, Keane T: **The Operating Characteristics of the PTSD Checklist in Detecting PTSD in HIV+ Substance Abusers**. *Journal of Psychological Trauma* 2008, **7**(4):213-234.

12. Hudson SA, Beckford LA, Jackson SD, Philpot MP: **Validation of a screening instrument for post-traumatic stress disorder in a clinical sample of older adults**. *Aging & mental health* 2008, **12**(5):670-673.

13. Keen SM: **Psychometric properties of PTSD Checklist in sample of male veterans**. *The Journal of Rehabilitation Research and Development* 2008, **45**(3):465-474.

14. Freedy JR, Steenkamp MM, Magruder KM, Yeager DE, Zoller JS, Hueston WJ, Carek PJ: **Post-traumatic stress disorder screening test performance in civilian primary care**. *Family practice* 2010, **27**(6):615-624.

15. Sydney Chiu, Webber MP, Zeig-Owens R, Gustave J, Lee R, Kelly KJ, Rizzotto L, McWilliams, Schorr JK, North CS *et al*: **Performance characteristics of the PtsD Checklist in retired firefighters exposed to the World trade Center disaster**. *Annals of Clinical Psychiatry* 2011, **23**(2):95-104.

16. Pietrzak RH, Van Ness PH, Fried TR, Galea S, Norris F: **Diagnostic utility and factor structure of the PTSD Checklist in older adults**. *International psychogeriatrics / IPA* 2012, **24**(10):1684-1696.

17. Gardner PJ, Knittel-Keren D, Gomez M: **The Posttraumatic Stress Disorder Checklist as a screening measure for posttraumatic stress disorder in rehabilitation after burn injuries**. *Archives of physical medicine and rehabilitation* 2012, **93**(4):623-628.

18. Karstoft KI, Andersen SB, Bertelsen M, Madsen T: **Diagnostic accuracy of the posttraumatic stress disorder checklist-civilian version in a representative military sample**. *Psychological Assessment* 2014, **26**(1):321-325.
