# Supplementary material for: Validity of the posttraumatic stress disorders (PTSD) checklist in pregnant women
Source: BMC Psychiatry. 2017 May 12;17:179. doi: 10.1186/s12888-017-1304-4 (PMC5427611; doi:10.1186/s12888-017-1304-4)
Supplement: Supplementary file 2 — Distribution of PCL-C scores according to clinician diagnosed PTSD status. (DOCX 20 kb) [file 12888_2017_1304_MOESM2_ESM.docx]

**Additional file 2: Figure S1. Distribution of PCL-C Scores According to Clinician Diagnosed PTSD Status**

Box plots comparing PCL-C scores among those classified as having PTSD (right side) and those without PTSD (left side) based on psychologist diagnosed PTSD using the CAPS. The central box shows the data between the upper and lower quartiles, with median represented by the middle line. The lines on either side of central box extend from the upper and lower quartiles to a distance of 1.5 × IQR (interquartile range) away or the most extreme data point within that range, whichever is smaller.
