# Supplementary material for: Validity of the posttraumatic stress disorders (PTSD) checklist in pregnant women
Source: BMC Psychiatry. 2017 May 12;17:179. doi: 10.1186/s12888-017-1304-4 (PMC5427611; doi:10.1186/s12888-017-1304-4)
Supplement: Supplementary file 3 — Receiver Operating Characteristic (ROC) Curves of PCL-C Score. (DOCX 23 kb) [file 12888_2017_1304_MOESM3_ESM.docx]

**Additional file 3: Figure S2. Receiver Operating Characteristic (ROC) Curves of PCL-C Score**
