# Supplementary material for: Validity of the posttraumatic stress disorders (PTSD) checklist in pregnant women
Source: BMC Psychiatry. 2017 May 12;17:179. doi: 10.1186/s12888-017-1304-4 (PMC5427611; doi:10.1186/s12888-017-1304-4)
Supplement: Supplementary file 4 — Sensitivity and specificity for PTSD diagnosis across various cut-off scores of Posttraumatic Stress Disorders Checklist Civilian Version (PCL-C) among subjects with lifetime sexual or physical abuse by intimate partner. (DOCX 17 kb) [file 12888_2017_1304_MOESM4_ESM.docx]

**Additional file 4: Table S3. Sensitivity and Specificity for PTSD Diagnosis across Various Cut-off Scores of Posttraumatic Stress Disorders Checklist Civilian Version (PCL-C) among Subjects With Lifetime Sexual or Physical Abuse by Intimate Partner**

| **Cut-off Score** | **Sensitivity**  **(95% CI)** | **Specificity**  **(95% CI)** | **Youden index** | **PPV (95% CI)** | **NPV (95% CI)** | **+LR (95% CI)** | **-LR (95% CI)** | **PR** |
| --- | --- | --- | --- | --- | --- | --- | --- | --- |
| 20 | 1.00 (0.96, 1.00) | 0.12 (0.12, 0.10) | 0.12 | 0.08 (0.07, 0.10) | 1.00 (0.97, 1.00) | 1.13 (1.10, 1.16) | 0.05 (0.00, 0.77) | 0.89 |
| 22 | 1.00 (0.96, 1.00) | 0.26 (0.23, 0.29) | 0.26 | 0.09 (0.07, 0.11) | 1.00 (0.99, 1.00) | 1.34 (1.29, 1.39) | 0.02 (0.00, 0.37) | 0.76 |
| 24 | 1.00 (0.96, 1.00) | 0.38 (0.35, 0.41) | 0.38 | 0.10 (0.08, 0.13) | 1.00 (0.99, 1.00) | 1.59 (1.52, 1.67) | 0.02 (0.00, 0.26) | 0.65 |
| **26** | **1.00 (0.96, 1.00)** | **0.48 (0.45, 0.51)** | **0.48** | **0.12 (0.10, 0.15)** | **1.00 (0.99, 1.00)** | **1.89 (1.78, 2.01)** | **0.01 (0.00, 0.21)** | **0.56** |
| **27** | **1.00 (0.96, 1.00)** | **0.52 (0.49, 0.55)** | **0.52** | **0.13 (0.11, 0.16)** | **1.00 (0.99, 1.00)** | **2.08 (1.95, 2.22)** | **0.01 (0.00, 0.19)** | **0.51** |
| 28 | 0.75 (0.64, 0.54) | 0.55 (0.52, 0.58) | 0.30 | 0.11 (0.08, 0.14) | 0.97 (0.95, 0.98) | 1.65 (1.43, 1.90) | 0.46 (0.31, 0.67) | 0.48 |
| 30 | 0.61 (0.49, 0.71) | 0.61 (0.58, 0.64) | 0.22 | 0.10 (0.08, 0.13) | 0.96 (0.94, 0.97) | 1.67 (1.29, 1.89) | 0.65 (0.49, 0.85) | 0.40 |
| 35 | 0.46 (0.35, 0.58) | 0.78 (0.75, 0.80) | 0.24 | 0.13 (0.10, 0.18) | 0.95 (0.94, 0.96) | 2.07 (1.60, 2.68) | 0.69 (0.56, 0.85) | 0.24 |
| 40 | 0.33 (0.23, 0.44) | 0.85 (0.82, 0.87) | 0.18 | 0.14 (0.09, 0.20) | 0.94 (0.93, 0.96) | 2.13 (1.51, 2.99) | 0.80 (0.68, 0.93) | 0.17 |
| 45 | 0.18 (0.11, 0.28) | 0.90 (0.88, 0.92) | 0.08 | 0.12 (0.07, 0.19) | 0.94 (0.92, 0.95) | 1.82 (1.11, 2.98) | 0.91 (0.82, 1.01) | 0.10 |

Abbreviations: PPV, positive predicted value; NPV, negative predicted value; +LR, positive likelihood ratio; -LR, negative likelihood ratio; PR: prevalence
