# Supplementary material for: Validity of the posttraumatic stress disorders (PTSD) checklist in pregnant women
Source: BMC Psychiatry. 2017 May 12;17:179. doi: 10.1186/s12888-017-1304-4 (PMC5427611; doi:10.1186/s12888-017-1304-4)
Supplement: Supplementary file 5 — Sensitivity and specificity for PTSD diagnosis across various cut-off scores of Posttraumatic Stress Disorders Checklist Civilian Version (PCL-C) among subjects without lifetime sexual or physical abuse by intimate partner. (DOCX 17 kb) [file 12888_2017_1304_MOESM5_ESM.docx]

**Additional file 5: Table S4. Sensitivity and Specificity for PTSD Diagnosis across Various Cut-off Scores of Posttraumatic Stress Disorders Checklist Civilian Version (PCL-C) among Subjects Without Lifetime Sexual or Physical Abuse by Intimate Partner**

| **Cut-off Score** | **Sensitivity**  **(95% CI)** | **Specificity**  **(95% CI)** | **Youden index** | **+PV (95% CI)** | **-PV (95% CI)** | **+LR (95% CI)** | **-LR (95% CI)** | **PR** |
| --- | --- | --- | --- | --- | --- | --- | --- | --- |
| 20 | 0.83 (0.64, 0.94) | 0.29 (0.27, 0.31) | 0.12 | 0.02 (0.01, 0.02) | 0.99 (0.98, 1.00) | 1.17 (0.99, 1.39) | 0.59 (0.26, 1.31) | 0.71 |
| **21** | **0.86 (0.68, 0.96)** | **0.40 (0.38, 0.42)** | **0.26** | **0.02 (0.01, 0.03)** | **1.00 (0.99, 1.00)** | **1.44 (1.24, 1.67)** | **0.34 (0.14, 0.85)** | **0.60** |
| 22 | 0.69 (0.49, 0.85) | 0.49 (0.47, 0.51) | 0.18 | 0.02 (0.01, 0.03) | 0.99 (0.98, 1.00) | 1.35 (1.05, 1.73) | 0.63 (0.37, 1.09) | 0.51 |
| 24 | 0.52 (0.33, 0.71) | 0.63 (0.60, 0.65) | 0.15 | 0.02 (0.01, 0.03) | 0.99 (0.98, 0.99) | 1.38 (0.97, 1.97) | 0.77 (0.53, 1.13) | 0.38 |
| **26** | **0.52 (0.33, 0.71)** | **0.72 (0.70, 0.74)** | **0.24** | **0.03 (0.01, 0.04)** | **0.99 (0.98, 1.00)** | **1.85 (1.29, 2.64)** | **0.67 (0.46, 0.98)** | **0.28** |
| 28 | 0.38 (0.21, 0.58) | 0.79 (0.77, 0.81) | 0.17 | 0.03 (0.01, 0.04) | 0.99 (0.98, 0.99) | 1.79 (1.12, 2.88) | 0.79 (0.77, 0.81) | 0.21 |

Abbreviations: +PV, positive predicted value; -PV, negative predicted value; +LR, positive likelihood ratio; -LR, negative likelihood ratio; PR: prevalence
