# Supplementary material for: Validity of the posttraumatic stress disorders (PTSD) checklist in pregnant women
Source: BMC Psychiatry. 2017 May 12;17:179. doi: 10.1186/s12888-017-1304-4 (PMC5427611; doi:10.1186/s12888-017-1304-4)
Supplement: Supplementary file 6 — Item mapping for confirmatory factor analysis. (DOCX 16 kb) [file 12888_2017_1304_MOESM6_ESM.docx]

**Additional file 6: Table S1. Item Mapping for Confirmatory Factor Analysis**

| **Item** | **Model** | | | |
| --- | --- | --- | --- | --- |
|  | 1 | 2 | 3 | 4 |
| 1. Memories/thoughts/images | R, A | R | R | R |
| 2. Repeated, disturbing dreams | R, A | R | R | R |
| 3. Acting/feeling as if happening again | R, A | R | R | R |
| 4. Feeling upset at reminders | R, A | R | R | R |
| 5. Physical reactions at reminders | R, A | R | R | R |
| 6. Avoid thinking/talking/feelings | R, A | A, N | A | A |
| 7. Avoid activities/situations | R, A | A, N | A | A |
| 8. Trouble remember details | R, A | A, N | N | D |
| 9. Loss of interest in activities | D | A, N | N | D |
| 10. Feeling distant or cut off from others | D | A, N | N | D |
| 11. Feeling emotionally numb | D | A, N | N | D |
| 12. Feeling as if future cut short | D | H | N | D |
| 13. Trouble sleeping | D | H | H | D |
| 14. Irritability/angry outbursts | H | H | H | D |
| 15. Difficulty concentrating | D | H | H | D |
| 16. Super alert or watchful | H | H | H | H |
| 17. Jumpy or easily started | H | H | H | H |

Abbreviations: R = re-experiencing, A = avoidance, N = numbing, H = hyperarousal, D = dysphoria

Model 1: model from EFA analysis; Models 2-4- identified from previous studies that underwent CFA
